# Adenomyoepithelioma of the Breast: A Case Report

**DOI:** 10.7759/cureus.68812

**Published:** 2024-09-06

**Authors:** Ioannis Rellias, Kallirroi Spanou

**Affiliations:** 1 Breast Surgery, Athens Euroclinic, Athens, GRC; 2 Breast Unit, Department of Obstetrics and Gynecology, National and Kapodistrian University of Athens, Alexandra General Hospital, Athens, GRC; 3 Pathology, Athens Euroclinic, Athens, GRC; 4 Pathology, Private Pathology Laboratory Pathlabs, Athens, GRC

**Keywords:** adenomyoepithelial tumor, benign, breast adenomyoepithelioma, rare breast tumor, uncommon breast tumor, well-circumscribed mass

## Abstract

Adenomyoepithelioma (AME) is an uncommon breast tumor distinguished by the presence of both epithelial and myoepithelial cell proliferation. It often presents clinically as a well-circumscribed, non-painful mass, although it can also be asymptomatic and discovered incidentally during imaging. This case report describes a 32-year-old woman with a tumor that progressively increased in size. It was initially assessed as a fibroadenoma based on ultrasonography and MRI, as the patient declined to undergo a core needle biopsy. The tumor poses significant diagnostic challenges due to its diverse imaging characteristics, necessitating a core needle biopsy for initial identification. There is also considerable variability within different regions of the same tumor, and surgical removal is typically recommended for most cases of AME. Most AMEs are benign, but they have the potential for local recurrence after surgical excision and, in rare cases, can become malignant. Accurate diagnosis and appropriate management can be achieved through clinical suspicion during examination, combined with the use of radiological techniques and histopathological analysis.

## Introduction

Adenomyoepithelioma (AME) is a rare breast tumor characterized by dual proliferation of epithelial and myoepithelial cells. To date, apart from case reports, only a few comprehensive series of studies have been documented [1-5]. In clinical examination, breast AME often presents as a well-defined, non-tender lump, although it may also be asymptomatic and discovered incidentally during imaging studies [2]. The majority of AMEs are benign, though they can recur locally after surgical excision and rarely may transform into a malignant form. Malignant transformation can involve one or both cellular components [3, 6-8]. These lesions can be difficult to diagnose, even with a core needle biopsy, due to their heterogeneity and variability across different regions of the same tumor. [9]. Surgical excision is recommended for managing AMEs to obtain a definitive histopathologic diagnosis and rule out malignancy [10].

Here, we report a rare case involving a 32-year-old female who was monitored with breast ultrasound and magnetic resonance imaging (MRI) over a two-year period for a lesion initially assessed as probably benign, possible fibroadenoma. Despite repeatedly declining a core needle biopsy, she eventually underwent a lumpectomy, which confirmed the diagnosis of a benign AME. 

## Case presentation

**Figure 1:**
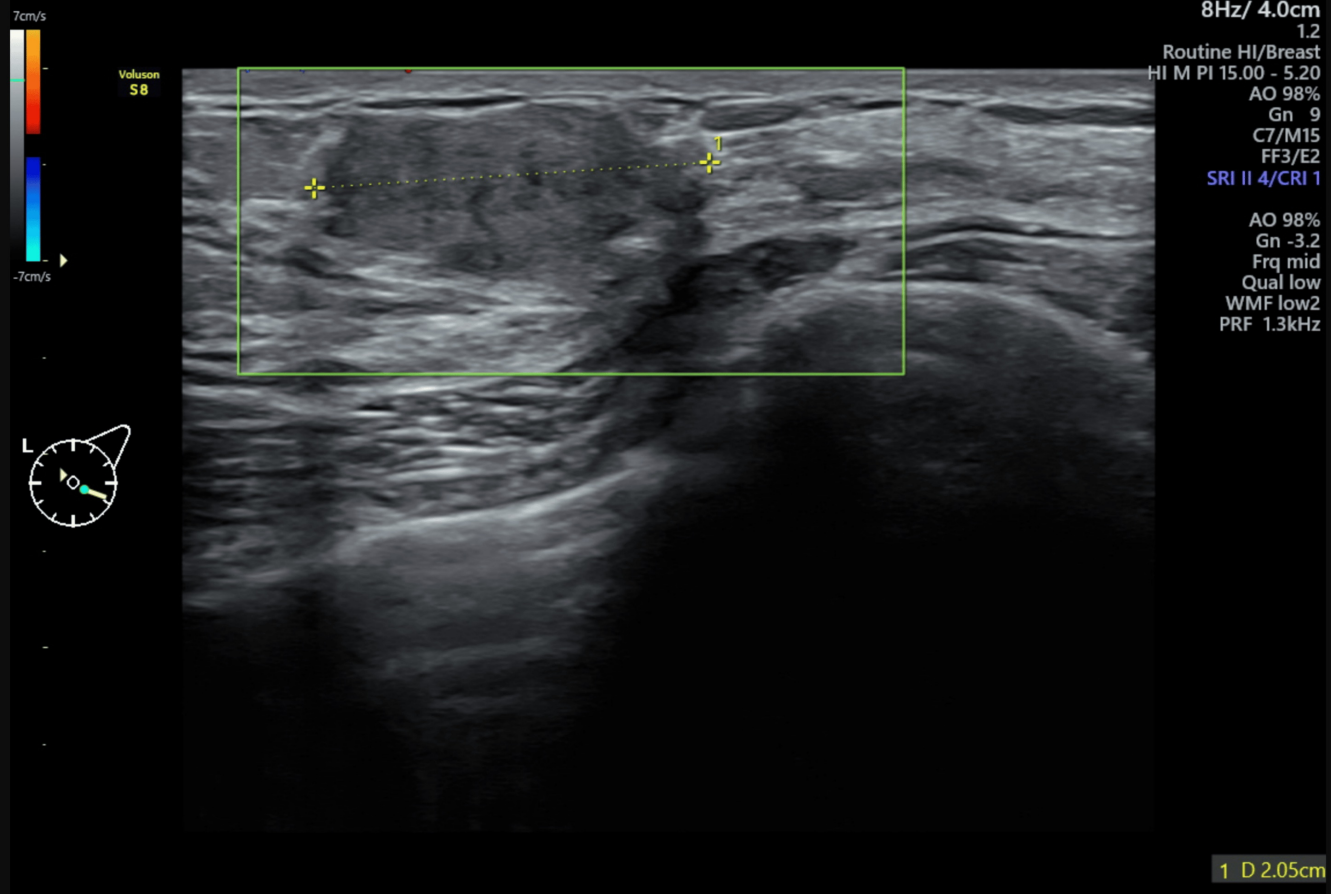
Ultrasound appearance of the adenomyoepithelioma A well-circumscribed hypoechoic lesion with a maximum diameter of 2.1 cm, lacking vascularity, and oriented parallel to the skin was noted.

A 32-year-old woman presented with a palpable, well-defined, non-tender, mobile mass in her left breast, located at the four o’clock position, 3 cm from the nipple, with a maximum diameter of 2.1 cm. Sonographic examination revealed the mass to have likely benign features: a well-circumscribed hypoechoic lesion, lacking vascularity, and oriented parallel to the skin (Figure 1).

Notably, her family history included a maternal grandmother who was diagnosed with breast cancer at the age of 45.

She was recommended to undergo a core needle biopsy of the finding but declined, choosing instead to proceed with further imaging using MRI. The MRI revealed a Breast Imaging-Reporting and Data System (BI-RADS) 3 finding in her left breast, and she decided on ultrasonographic follow-ups every six months. Subsequent examinations noted a slight increase in mass size (from 2.1 cm to 2.4 cm over a year), along with increased peripheral vascularity. She was again advised to have a core needle or excisional biopsy but refused.

**Figure 2:**
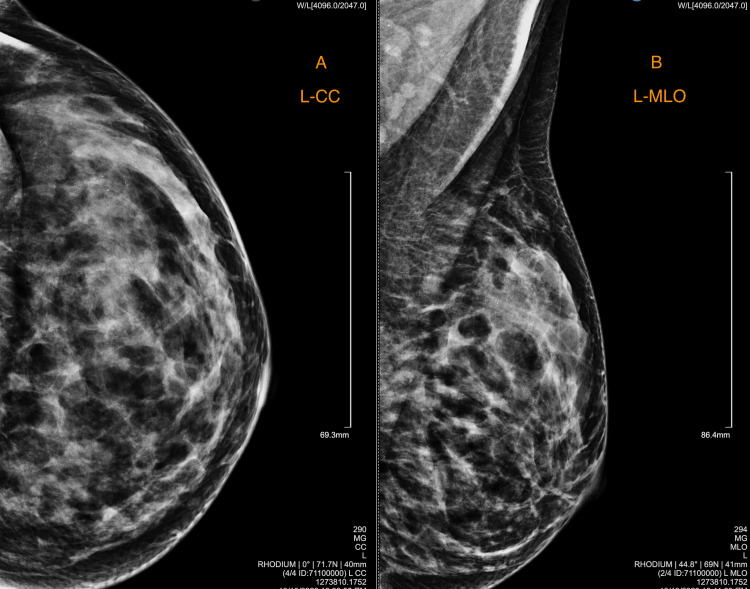
Digital mammography showed dense breasts without any suspicious findings. (Α) Left breast, craniocaudal view (L-CC) and (B) Left breast, mediolateral oblique view (L-MLO)

She returned nearly a year later, and we discovered a well-defined hypoechoic mass with a maximum diameter of 2.4 cm, characterized by lobular margins and peripheral vascularity. Digital mammography indicated dense breasts without any suspicious findings (Figure 2).

**Figure 3:**
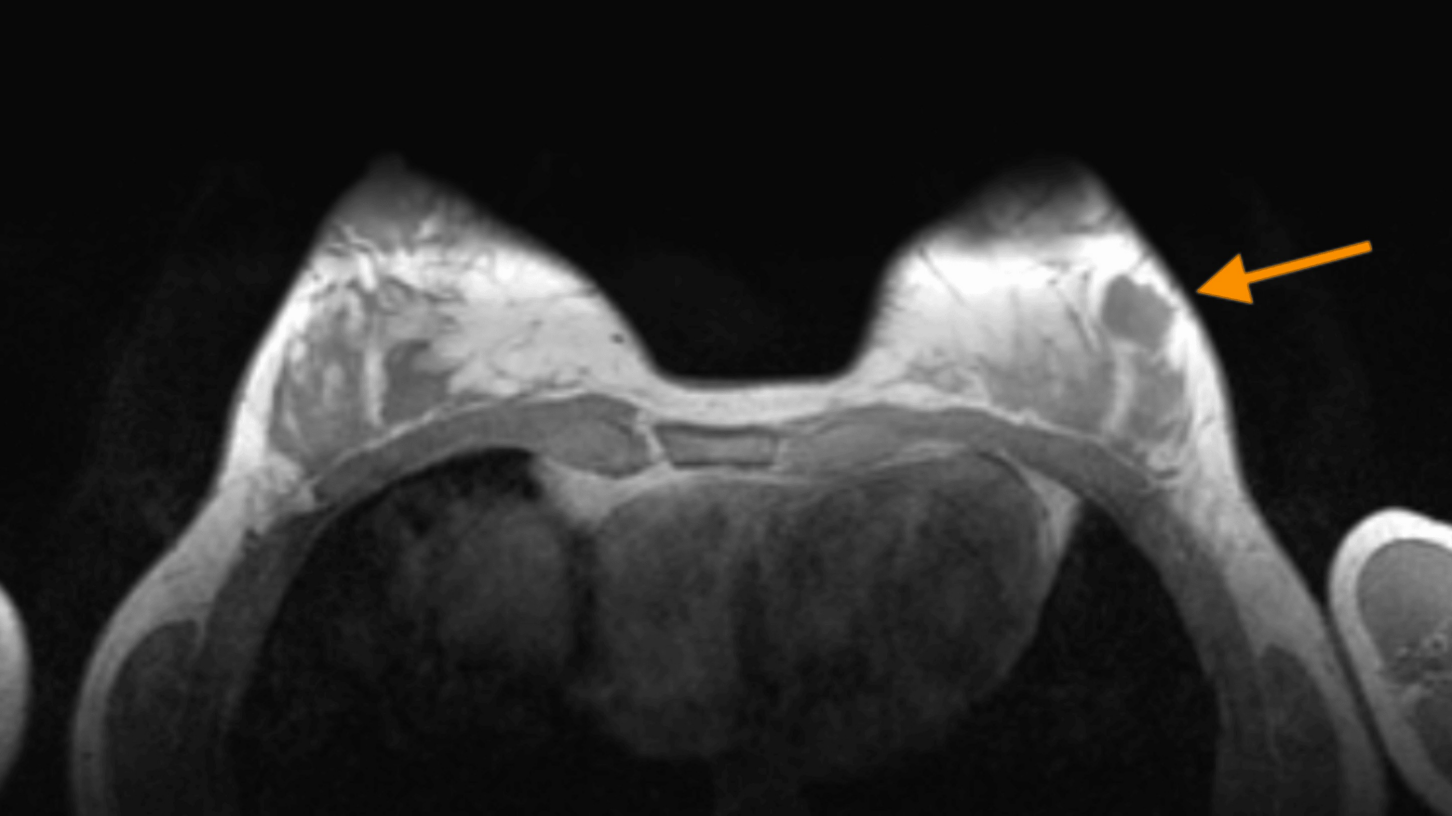
The MRI (T1WI) showed a solid lesion with lobular margins located at the four o’clock position of the left breast (arrow) showing hypointensity on T1WIs. MRI: magnetic resonance imaging; T1WI: T1-weighted images

**Figure 4:**
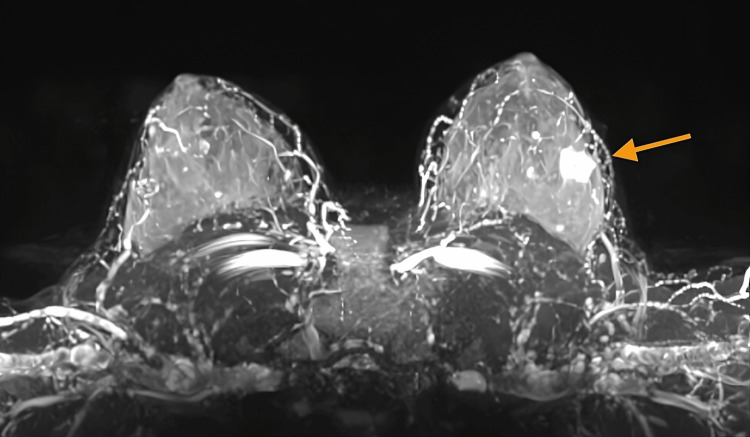
The MRI (T2WI-3D) showed a solid lesion with lobular margins located at the four o’clock position of the left breast (arrow), showing hyperintensity on T2WIs MRI: magnetic resonance imaging; T2WI: T2-weighted image; 3D: three dimensional remodeling

**Figure 5:**
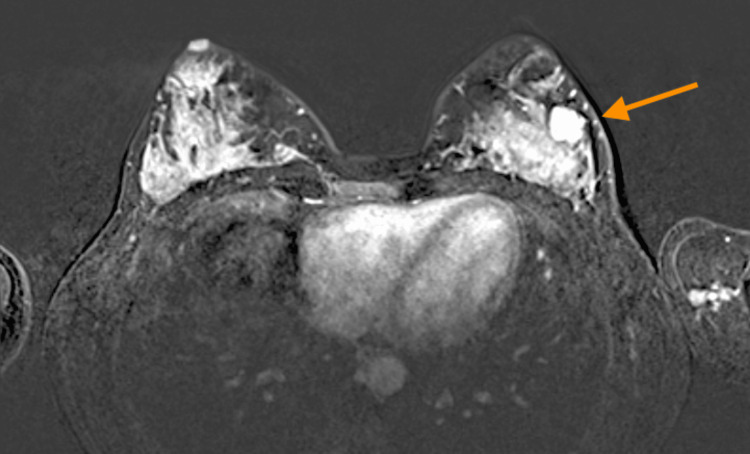
The MRI showed a mass (arrow) with early enhancement after paramagnetic contrast administration. MRI: magnetic resonance imaging

The MRI revealed a left breast solid lesion with lobular margins located at the four o’clock position, showing changes in the diffusion sequences compared to the MRI conducted two years earlier, and classified the lesion as BI-RADS 4A (Figures 3-5). 

**Figure 6:**
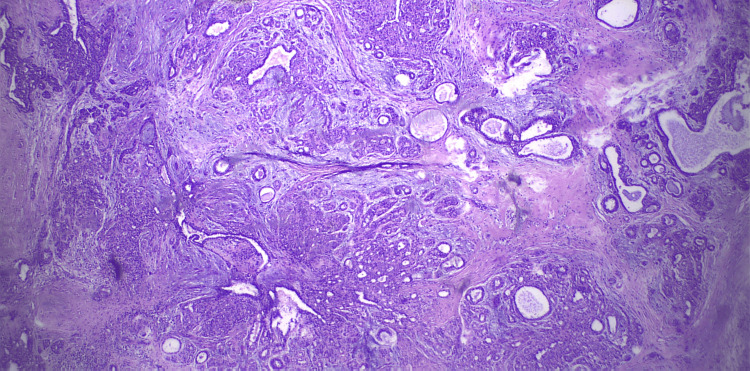
The H&E stain in low magnification view demonstrated a biphasic proliferation composed of a myoepithelial cell proliferation surrounding epithelial glands. H&E stain: hematoxylin and eosin stain

**Figure 7:**
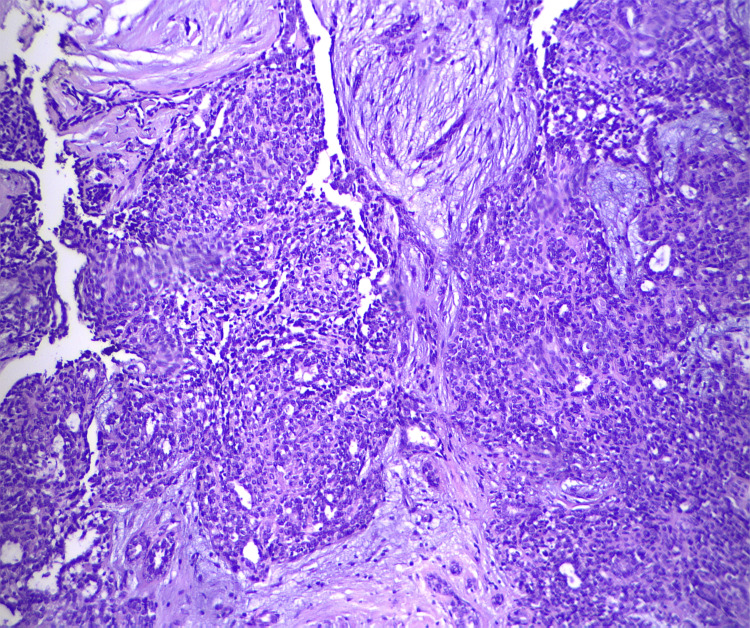
The H&E stain in higher power image demonstrated the dual cell population of myoepithelial cells surrounding epithelial glands. H&E stain: hematoxylin and eosin stain

**Figure 8:**
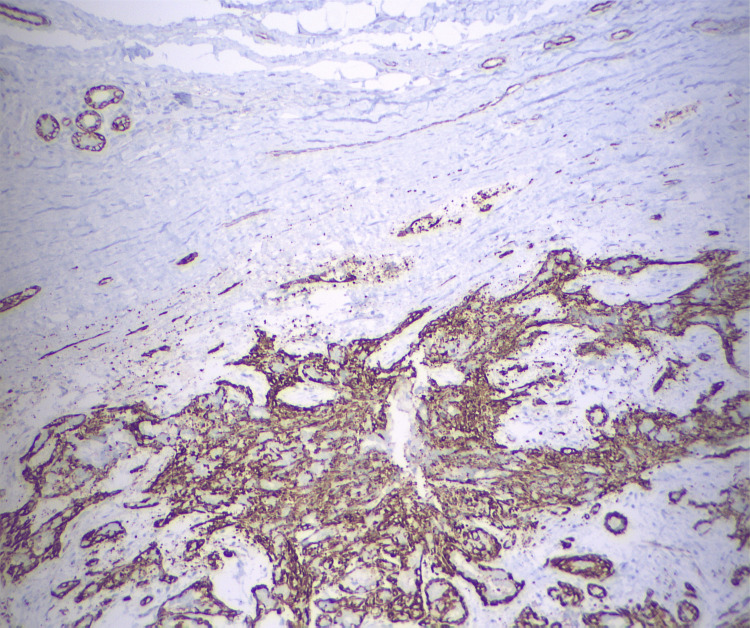
The myosin immunohistochemical stain highlighted the myoepithelial cells in this lesion, surrounding the epithelial proliferation. H&E stain: hematoxylin and eosin stain

A core needle biopsy was recommended, but the patient declined, opting instead for an excisional biopsy of the tumor, which led to a histological diagnosis of AME (Figures 6-8).

## Discussion

Adenomyoepithelioma is a rare breast tumor that may pose both clinical and diagnostic challenges. It was first described by Hamperl in 1970 [11].

Histologically, it consists of a biphasic proliferation of myoepithelial and epithelial cells. Adenomyoepithelioma lesions are classified into three types: tubular, lobulated, or spindle cell variants, with these growth patterns occasionally coexisting [2]. The predominant papillary architecture of the tumor in most cases supports the theory that it may originate from an intraductal papilloma [1]. The median age at presentation is reported to be 67 years, although it can occur at any age [4].

Typically, it presents as a single breast mass, with the average tumor size being 2 to 2.5 cm [4]. Most are benign and may recur locally, but malignant transformation is possible [12,13]. When there is rapid growth, it strongly indicates malignant transformation. The associated carcinomas can be of low or high grade and may metastasize to the lungs, bone, brain, or liver [8, 14, 15].

Diagnosis can be complex and challenging, due to heterogeneity and variability [9]. The radiological images are non-specific. On ultrasound, it may appear as an oval or circumscribed hypoechoic mass, but sometimes it may have irregular or microlobulated margins. Color Doppler ultrasound can also be utilized to delineate lesion vascularity [16]. On mammography, AME appears as a round, oval, or lobulated high-density mass with well-defined borders, occasionally showing areas with indistinct margins [17]. The size varies from 0.3 to 7 cm, with an average diameter of 2.5 cm. Larger sizes and irregular borders may indicate malignancy. Microcalcifications are uncommon but have been observed, and they are associated with a poorer prognosis [4, 18]. On MRI, AME typically appears as an isointense mass on T1-weighted images (T1WI) and shows hyperintensity on T2-weighted images (T2WI). This mass often exhibits a homogeneous progressive enhancement or a heterogeneous enhancement with either washout or plateau enhancement kinetics [5, 17].

The histological diagnosis of AME can also be challenging. Cytological examination alone often leads to confusion with other types of neoplasms. However, core biopsy provides more accurate and detailed information. Key diagnostic features include tightly packed glands forming compact nodules and the presence of prominent clear cell or spindle cell myoepithelium. Immunohistochemical staining for myoepithelial markers is beneficial for identifying the myoepithelial component [19]. A complete excision is required to thoroughly assess for atypia or carcinoma development within an AME.

The primary treatment involves a wide local excision surgery, ensuring clear margins to prevent local recurrence. Axillary staging is required only in cases of malignant transformation [20].

## Conclusions

Breast AME is an uncommon tumor that can present considerable diagnostic challenges due to its varied imaging features and the need for a core needle biopsy for initial identification. An accurate diagnosis and effective treatment plan can be attained by maintaining clinical suspicion during the examination, along with utilizing radiological imaging and histopathological evaluation. This case underscores the importance of considering AME in the differential diagnosis of both symptomatic and asymptomatic breast masses. Wide local excision is typically recommended for most cases of AME, knowing that there is also considerable variability within different regions of the same tumor; it can recur locally and, in rare cases, may become malignant. Further research on the tumor's development and its long-term outcomes following wide surgical excision is necessary to inform future treatment strategies.
